# Perturbations to the IGF1 growth pathway and adult energy homeostasis following disruption of mouse chromosome 12 imprinting

**DOI:** 10.1111/apha.12160

**Published:** 2013-09-18

**Authors:** M Charalambous, S T da Rocha, A Hernandez, A C Ferguson-Smith

**Affiliations:** 1 Department of Physiology, Development and Neuroscience, University of CambridgeCambridge, UK; 2 Maine Medical Center Research InstituteScarborough, ME, USA

**Keywords:** growth, IGF1 signalling, imprinting

## Abstract

**Aim:**

Disruption to insulin-like growth factor (IGF) signalling pathways during early life causes growth retardation and defects of developing metabolic organs that can alter set points of energy homeostasis for a lifetime. Inheritance of two maternal copies of human chromosome 14q32.2 (Temple syndrome) causes severe foetal growth retardation and post-natal failure to thrive. Disruption of imprinted gene dosage in the orthologous region on mouse chromosome 12 also affects growth. Here, we investigated whether altering chromosome 12-imprinted gene dosage can affect IGF signalling.

**Methods:**

We investigated mice with a transgene insertion at the imprinted domain of chromosome 12. This lesion causes misexpression of neighbouring genes such that the expression of non-coding RNAs is elevated, and levels of delta-like homologue 1 (*Dlk1*), retrotransposon-like 1 (*Rtl1*) and deiodinase 3 (*Dio3*) transcripts are reduced.

**Results:**

We observed three key phenotypes in these mice: (i) embryonic growth retardation associated with altered expression of IGF1 binding proteins, (ii) peri-natal failure to thrive accompanied by hypothyroidism and low serum IGF1. Unexpectedly this phenotype was growth hormone independent. (iii) Adult animals had reduced glucose tolerance as a result of endocrine pancreatic insufficiency.

**Conclusions:**

We propose that all of these phenotypes are attributable to impaired IGF action and show for the first time that the chromosome 12 cluster in the mouse is an imprinted locus that modulates the IGF signalling pathway. We propose that growth retardation observed in human Temple syndrome might have a similar cause.

Infants born small as a result of foetal growth restriction (FGR) have a peri-natal mortality rate approx. 10-fold higher than those born at appropriate weight for gestational age (Randhawa & Cohen 2005). The insulin-like growth factor (IGF) axis plays a critical role in foetal and post-natal growth. In mice and humans, mutations in IGF signalling components cause FGR (reviewed in Efstratiadis 1998, Rodriguez *et al*. 2007). However, despite the observation that serum IGFs are low in small-for-gestational-age newborns, only a small proportion have mutations in genes of the core IGF pathway (Randhawa & Cohen 2005). Thus, further modulators of IGF signalling must exist to explain these idiopathic cases of FGR.

Pituitary growth hormone (GH) is a major modulator of IGF1 levels, and individuals with congenital GH deficiency (e.g. Laron syndrome) or hypopituitarism are growth-retarded in the third trimester with reduced IGF1 at birth (Randhawa & Cohen 2005). The major role for GH is in post-natal growth, and the post-pubertal growth spurt is mediated by hepatic secretion of IGF1 (Mathews *et al*. 1988). In the mouse, GH does not drive intra-uterine growth, and growth retardation phenotypes are not observed in genetic models of impaired GH signalling until after post-natal day 10 (e.g. Lupu *et al*. 2001). In both mice and humans, IGF1 and IGF2 are the major foetal growth factors, and they exert a continuous role on growth throughout development (Efstratiadis 1998, Klammt *et al*. 2008). The relative delay of GH signalling in the mouse makes it a useful model in which to study GH and IGF signalling independently, because any growth effects observed in late gestation and early post-natal development in the mouse cannot be a consequence of an impaired GH pathway.

After birth, a significant proportion of growth-restricted infants suffer various childhood morbidities including poor growth and intellectual impairment (Klammt *et al*. 2008). Moreover, several adult chronic diseases (including reduced glucose tolerance, obesity, type II diabetes and hypertension) are more common in individuals with FGR (Barker *et al*. 1993). Impaired IGF signalling is associated with metabolic risk traits (reviewed in Rodriguez *et al*. 2007). This is because, in addition to their important role in longitudinal growth, IGFs are necessary for the development of post-natal organ systems that regulate energy homeostasis (reviewed in Kawai & Rosen 2010). For example, *Igf1 receptor* (*Igf1r*) expression is necessary for the late post-natal development of the endocrine pancreas, and deletion of this gene prevents islet formation (Withers *et al*. 1999). Less severe disruption of the IGF axis in the pancreas causes failures of insulin secretion and glucose tolerance (Withers *et al*. 1999, Kulkarni *et al*. 2002).The IGF pathway therefore links foetal growth and adult onset metabolic disease.

Several important paediatric disorders associated with FGR and post-natal failure to thrive are caused by uniparental disomies of critical regions of the genome. These critical regions contain imprinted genes, a group of approx. 100 genes whose expression is dependent upon parental origin. These genes typically occur in clusters, and uniparental inheritance of imprinted regions causes loss or gain of expression of multiple genes (reviewed in Yamazawa *et al*. 2010). Correct imprinted gene dosage is necessary for normal intra-uterine growth and placentation, as well as to maintain energy homeostasis in adult life (reviewed in Charalambous *et al*. 2007). Many components of the IGF signalling pathway are regulated by imprinting, either directly or indirectly. *Igf2,* which encodes a potent embryonic growth factor, is expressed predominantly from the paternally inherited copy of mouse chromosome 7 (DeChiara *et al*. 1991, Ferguson-Smith *et al*. 1991), and overexpression by biallelic expression causes overgrowth in mice (Leighton *et al*. 1995, Sun *et al*. 1997) and Beckwith–Wiedemann syndrome in humans (Yamazawa *et al*. 2010). Loss of *Igf2* function results in severe embryonic growth retardation in mice (DeChiara *et al*. 1990) and is one of the lesions underlying Silver–Russell syndrome in humans. *Igf2* binds the Igf2/mannose-6-phosphate receptor (*Igf2r*), which causes degradation of the ligand. *Igf2r* is imprinted and expressed from the maternally inherited chromosome in the mouse (Barlow *et al*. 1991). The maternally expressed *H19* transcript contains microRNA-675, a placental growth repressor whose depletion results in increased expression of the *Igf1r* gene (Keniry *et al*. 2012). Imprinted *Grb10* is a negative regulator of *Igf1r* signalling (Morrione *et al*. 1996). Deletion of *RasGrf1* from the paternal chromosome results in post-natal growth retardation and insensitivity to IGF1 (Itier *et al*. 1998, Clapcott *et al*. 2003, Font de Mora *et al*. 2003). Thus, imprinting acts on the IGF pathway by modulating expression dosage of the pathway components themselves and by poorly understood mechanisms that modify IGF action.

Paternal uniparental disomy of chromosome 14 causes Kagami syndrome, with placentomegaly, bell-shaped thorax and developmental retardation. Maternal uniparental disomy in this region cause Temple syndrome, which is characterized by severe intra-uterine growth restriction with initial failure to thrive, hypotonia, obesity and precocious puberty (reviewed in Hoffmann & Heller 2011). Loss of function and/or increased gene dosage of multiple genes are thought to contribute to the Kagami/Temple phenotypes (Ogata *et al*. 2008). The imprinted region on chromosome 14 and its orthologous region on mouse chromosome 12 contain several imprinted genes. Three protein-encoding genes, the delta-like homologue 1 (*Dlk1*), retrotransposon-like gene 1 (*Rtl1*) and deiodinase 3 (*Dio3*), are expressed from the paternally inherited chromosome and repressed on the maternally inherited chromosome. An array of functional non-coding RNAs (*Gtl2*, *Rtl1-antisense*, a large cluster of microRNAs *Mirg* and *CD/SnoRNAs*) are expressed from the maternally inherited chromosome (reviewed in da Rocha *et al*. 2008). Expression dosage of all of these genes is controlled by an imprinting control region, the intergenic differentially methylated region (IG-DMR; Lin *et al*. 2003). The IG-DMR contains a cluster of CpG dinucleotides that are methylated on the paternally inherited chromosome and unmethylated on the maternally inherited chromosome. These epigenetic marks are acquired during gametogenesis and maintained throughout development in somatic cells (Takada *et al*. 2000, 2002).

Maternal UPD12 in the mouse causes some of the clinical symptoms of Temple syndrome (notably growth retardation and muscular hypotonia). However, these animals die pre-natally, making further comparisons with the human syndrome impossible (Georgiades *et al*. 2000). Furthermore, the involvement of the IGF pathway in the growth retardation phenotype has not been explored. To evaluate the consequences of misexpression of the genes in the chromosome 12 imprinting cluster, we have made use of a mouse model of partial loss of imprinting, such that the normally paternally expressed genes are down-regulated and the maternally expressed genes are slightly activated when paternally inherited (Gtl2LacZ mice; Schuster-Gossler *et al*. 1996, Steshina *et al*. 2006). These animals are viable and fertile, exhibit mild intra-uterine growth restriction followed by severe failure to thrive. We show that in mice inheriting Gtl2LacZ insertion paternally, the IGF pathway is disrupted at multiple levels and that adult animals have compromised glucose homeostasis as a result of a defect in pancreatic function.

## Material and methods

### Breeding of transgenic animals

The Gtl2LacZ insertion was generated by Schuster-Gossler *et al*. (1996), and PCR genotyping was performed with the following primers: E20-3005F 5′ ATTCTCTGGTGCCCCCCGTT 3′, Gtl2-TRLacZ1 5′ AGCCACAGACGTCATTATGC 3′ and LacZ4 5′ CCAGATAACTGCGTCACTCC 3′. Paternal transmission Gtl2LacZ insertion mutants (TG^PAT^ animals) were generated from either TG^MAT^ or TG^PAT^ males crossed with 129Sv [wild-type (WT)] females. We did not observe significant differences in embryonic growth or post-natal phenotype between the two grand paternal types so these data are combined in this study. For the embryonic studies, the day of vaginal plug was considered day E0.5. As mutant animals were growth-retarded, we delayed weaning of all litters until the fourth post-natal week (P28–P30). Animals were housed at a density of 3–4 per cage in a temperature-controlled room (20–22 °C) with 12-h light/dark cycle. All experiments involving mice were carried out in accordance with UK Government Home Office licensing procedures.

### Methylation analysis

DNA methylation at the IG-DMR, Gtl2 promoter and Dlk1 DMR was performed using methylation-sensitive Southern blotting using probes and digests as reported previously (Takada *et al*. 2002). We quantified the intensity of the undigested product relative to the internal control to provide a ‘fully methylated’ value and plotted the intensity relative to the level observed in mUPD12 or pUPD12 DNA in which the parental chromosomes are hypo-or hypermethylated respectively. We quantified intensities of diagnostic and control bands on a Storm 860 phosphorimager using Amersham software (G. E. Healthcare, Buckinghamshire, UK).

### Expression studies

RNA was prepared from snap-frozen tissues using Trizol (Life Technologies Ltd, Paisley, UK) according to the manufacturer's instructions.

### Northern blotting

mRNA was extracted from 100 μg of total RNA Dynabeads Oligo (dT)_25_ kit (Life Technologies Ltd) following the supplied protocol and used 0.5 μg mRNA per sample in a standard Northern blotting protocol with probes complementary to *Dlk1* and *Gapdh*, as described previously (da Rocha *et al*. 2009). We quantified intensities of diagnostic and control bands on a Storm 860 phosphorimager using Amersham software, and the level of Dlk1 was normalized to the loading control Gapdh.

### RNase protection

The assay was performed essentially according to Isaacs *et al*. (1992). P32-labelled probes were generated by *in vitro* transcription from cloned fragments of *Rtl1* and *Rtl1AS* (between nucleotides 2444 and 2835, NM184109), *Dio3* (571 and 776, NM172119) and alpha tubulin (178 and 277, BC056169) using T7 (Promega, Southampton, UK) and SP6 polymerases (Life Technologies Ltd) according to the manufacturers' instructions. Yeast total RNA was used as a negative control did not generate a signal of the protected size with any of the probes. We quantified intensities of diagnostic and control bands on a Storm 860 phosphorimager using Amersham software, and the level of target genes was normalized to the loading control alpha tubulin.

### Real-time quantitative PCR

cDNA was generated from 2 μg total RNA, which had been treated with DNase I (Promega), using the RevertAid H Minus cDNA synthesis kit (Thermo Scientific, Leicestershire, UK) with random primers following the supplied protocol. Real-time quantitative PCR with SYBR Green was performed with SensiMix (Quantace, Bioline, London, UK) according to the manufacturer's instructions using the primers in Table S1. Quantification was performed using the relative standard curve method, and target gene expression was normalized to the expression of *Hprt*, the expression of which did not differ between the groups (not shown). All primers amplified with efficiency ≥85%.

### Phenotypic characterization of Gtl2LacZ transgenic animals

#### Serum biochemistry

Serum peptides were quantified by ELISA: IGF1 (rat/mouse IGF1 ELISA, IDS, using the suggested protocol to dissociate from IGF binding proteins), triiodothyronine and total thyroxine (TT3, TT4; Alpha Diagnostic International, San Antonio, TX, USA), GH (Merck, NJ, USA), leptin and insulin (CrystalChem, Downers Grove, IL, USA) all according to the manufacturer's instructions.

#### D3 assays

D3 activity was determined as described (Hernandez *et al*. 2006). A suitable volume of tissue homogenate was used in the enzymatic reaction to ensure that deiodination did not exceed 20% and was proportional to the amount of protein content.

#### Stereology

Whole pancreas was trimmed for adipose tissue, then weighed and fixed in 4% paraformaldehyde in three separate tissue preparations. After dehydration in ascending ethanol and wax, embedding serial sections were cut at 7 μm from each preparation and stained with haematoxylin and eosin. Endocrine and exocrine mass was estimated by point counting six randomly assigned fields of view per section and scoring for islet, acinus or duct. Mass was generated by multiplying the combined proportion by the total pancreatic mass. In addition, the surface areas of all islets in the fields of view were measured. Randomization, point counting and surface area measurements were performed using the Computer Assisted Stereology Toolbox (cast) 2.0 system from Olympus (Ballerup, Denmark).

#### Glucose and insulin tolerance tests

Glucose (GTT) and insulin tolerance tests were carried out on 6-month-old mice according to published protocols (Vidal-Puig *et al*. 2000).

### Statistical analysis

All statistical tests were performed using the GraphPad Prism Software, version 4.00, for Windows; GraphPad Software (San Diego, CA, USA, http://www.graphpad.com). Specific tests, significance values and number of samples analysed are indicated in the respective figure/table legends.

## Results

### Insertion of a LacZ transgene disrupts somatic DMR methylation and gene expression

To explore the function of the chromosome 12-imprinted gene cluster in growth pathways, we made use of the Gtl2LacZ transgenic model first described by Schuster-Gossler *et al*. (1996). This genetic model is the result of the integration of a 15-kb array, comprised of 2–3 copies of a promoter-less LacZ/β-neo transgene, 1.6 kb upstream of the *Gtl2* promoter (Paulsen *et al*. 2001). Other groups have reported the phenotype of paternal transmission of this transgene to be partially penetrant and dependent on genetic background (Schuster-Gossler *et al*. 1996, Steshina *et al*. 2006). With this in mind, we backcrossed the Gtl2LacZ mice onto a 129SvJ background. We then characterized methylation and gene expression in late gestation mouse embryos following paternal transmission of the Gtl2LacZ insertion (hereafter TG^PAT^) compared with their WT littermates.

**Figure 1:**
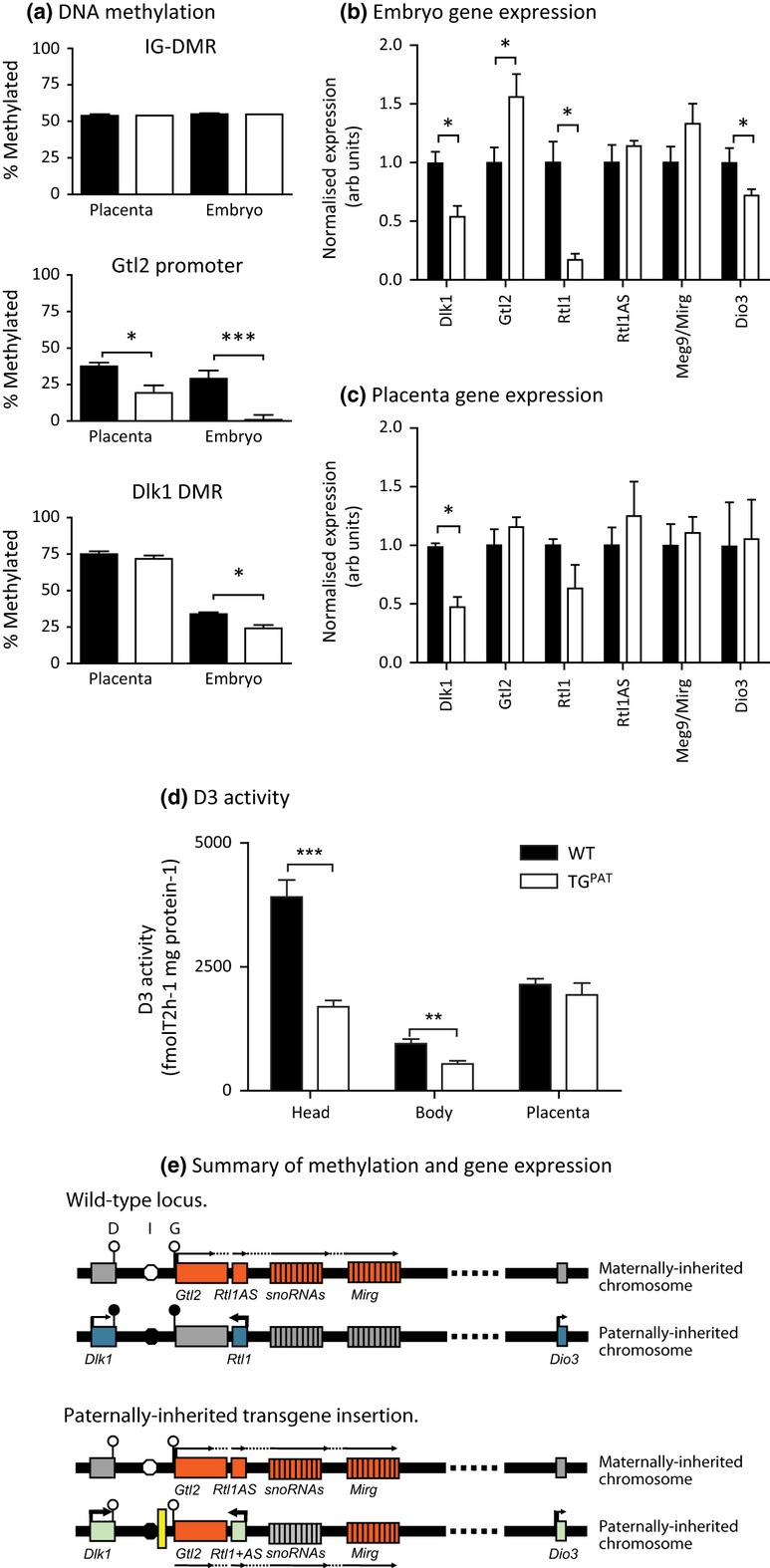
Paternal transmission of the Gtl2LacZ insertion disrupts methylation and gene expression in the Dlk1-Dio-imprinted cluster. (a) Levels of DNA methylation were measured at representative CpGs within the intergenic differentially methylated region (IG-DMR), Gtl2 promoter and Dlk1 DMR by methylation-sensitive Southern blotting. Methylation at the IG-DMR was unaffected by the transgene insertion. At the Gtl2 promoter, methylation was lost in the TG^PAT^ embryo (1.0 vs. 29.1%, *P* < 0.01, *n* = 7 per genotype) and placenta (19.3 vs. 37.5%, *P* < 0.01%, *n* ≥ 4 per genotype). Dlk1 DMR was reduced as a result of the transgene insertion in the embryo 24.2 vs. 33.9%, *P* < 0.05, *n* = 4 per genotype). *P*-values were assessed by Mann–Whitney *U*-test. (b, c) Expression dosage of chromosome 12-imprinted genes was measured in the e15.5 embryo (b) and placenta (c) by Northern blotting (Dlk1), RT-qPCR (Gtl2 and Mirg) and RNase protection (Rtl1, Rtl1AS and Dio3). *P*-values assessed by Mann–Whitney *U*-test. In the embryo, expression of all paternally expressed genes was reduced in the presence of the transgene insertion [Dlk1 54% (*P* < 0.05, *n* ≥ 4), Rtl1 17% (*P* < 0.05 *n* ≥ 4), Dio3 72% (*P* < 0.05, *n* ≥ 4 per genotype) of wild-type (WT) levels]. Expression from the maternally expressed non-coding RNAs was elevated [Gtl2 156% (*P* < 0.05, *n* = 8), Rtl1AS 114% (ns, *n* ≥ 4), Mirg 133% (ns, *n* = 8)] of WT levels but only significantly for Gtl2. In the placenta, only expression of Dlk1 was significantly affected by the transgene insertion (47% of WT levels, *P* < 0.05, *n* ≥ 4). (d) The enzymatic activity of deiodinase 3 (D3) was determined in head, body and placenta of conceptuses at e15.5. D3 activity was reduced in head and body (head 43% WT levels, *P* < 0.001; body 57% WT, *P* < 0.01) of TG^PAT^ embryos, but not in the placenta (90% WT, ns). *n* = 8 per genotype, genotypes were compared by Mann–Whitney *U*-test. (e) Representation of gene expression and methylation status at the imprinted region on chromosome 12, in WT animals and on paternal transmission of the Gtl2LacZ insertion. Genes normally expressed from the maternally inherited chromosome are shown in red and those expressed from the paternally inherited chromosome in blue. Grey boxes represent silenced genes. Light green boxes represent reduced expression upon Gtl2LacZ insertion. The three DMRs, the exon 5 Dlk1 DMR (D), the IG-DMR (I) and the Gtl2 promoter DMR (G) are shown as white (unmethylated) or black (methylated) circles. Yellow box indicates the position of the Gtl2LacZ insertion.

We measured DNA methylation at three known DMRs in the chromosome 12-imprinted domain (Takada *et al*. 2002), the IG-DMR, the *Gtl2* promoter and the exon 5 Dlk1 DMR (Fig. 1a). Consistent with previous reports, methylation at the IG-DMR was unaffected by paternal transmission of the transgene insertion. However, we observed complete loss of methylation of the *Gtl2* promoter in TG^PAT^ embryos, presumably due to loss of methylation *in cis* to the insertion, as previously reported (Sekita *et al*. 2006, Steshina *et al*. 2006). In the placenta, we observed a partial loss of methylation at this region. In addition, we could detect a small reduction in methylation at the *Dlk1* DMR in the embryo but not in the placenta.

Alterations to the methylation status of these regulatory regions were associated with changes to gene expression. In the embryo, expression of the paternally expressed genes was reduced, and levels of maternally expressed *Gtl2* were elevated (Fig. 1b). In the placenta, we observed a similar trend for reduced expression of paternally expressed genes and activation of maternally expressed genes, but this was only statistically significant for *Dlk1* (Fig. 1c).

We next asked whether the changes to *Dio3* gene expression were functionally relevant by performing assays for Deiodinase 3 (D3) enzymatic activity. We were able to show that D3 activity was reduced relative to WT in the e15.5 TG^PAT^ embryo (head and body, Fig. 1d) but not in the placenta, consistent with the gene expression data.

We concluded that in embryonic tissues, the insertion of the Gtl2LacZ transgene on the paternally inherited chromosome causes disruption of the somatic DMRs and concomitant de-repression of *Gtl2* and partial silencing of *Dlk1*, *Rtl1* and *Dio3* (summarized in Fig. 1e). In the placenta, perturbation of methylation was less severe, and gene expression changes were more moderate.

### TG^PAT^ conceptuses have reduced growth and disrupted expression of IGF pathway components

**Figure 2:**
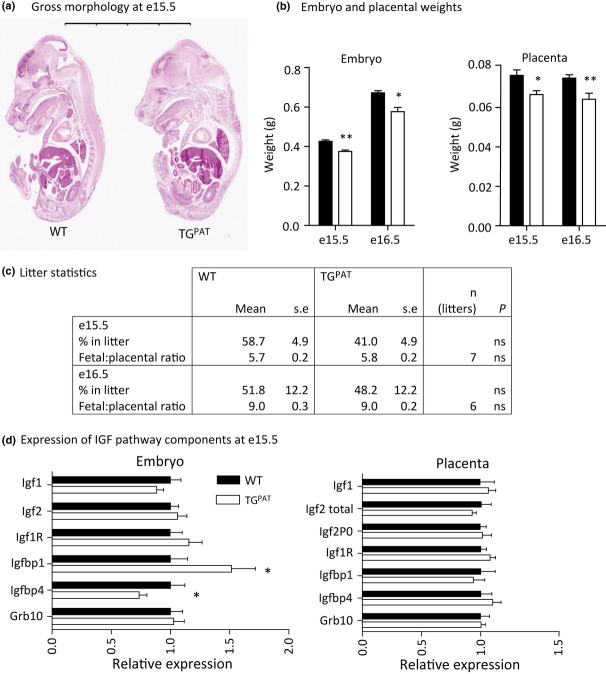
TG^PAT^ embryos are small with perturbed expression of insulin-like growth factor (IGF) pathway genes. (a) Haematoxylin and eosin staining of midline sagittal sections of e15.5 embryos. Scale bar shows 8 mm. (b) Embryonic and placental weights at e15.5 and e16.5 [e15.5 wild-type (WT) *n* = 30, TG^PAT^ *n* = 22 from seven litters; e16.5 WT *n* = 17, TG^PAT^ *n* = 24 from six litters]. Bars show litter means by genotype, ±SE. Genotypes were compared by Mann–Whitney *U*-test, *P < 0.05, **P < 0.01. (c) Descriptive statistics collected from seven litters of a +/+ × +/Gtl2LacZ cross at e15.5 and six litters of the same cross at e16.5. Deviation from a Mendelian ratio was tested using a Wilcoxon's signed rank test against a theoretical median of 50%. The foetal/placental mass ratio was calculated by dividing the foetal mass by the placental mass for each conceptus and is presented as the mean value ± the standard error of the mean (SE) for each genotype. (d) Gene expression of IGF pathway components as determined by quantitative real-time PCR on RNA isolated from whole embryos or placentae at e15.5. Data are normalized to WT values and expressed as mean relative expression ± SE. *n* = 8 samples per tissue per genotype, *P < 0.05 by Mann–Whitney *U*-test.

At e15.5, TG^PAT^ embryos were grossly morphologically normal (Fig. 2a), but were growth-restricted by approx. 12% in weight. Placental weight was reduced by approx. 14%. To ask whether the growth phenotype became more severe with developmental age, we measured foetal and placental weight 1 day later. At e16.5, the same level of growth restriction was observed (12% reduction in weight vs. WT littermates in both placenta and embryo, Fig. 2b), indicating that there was no increase in severity with age. At both ages, placental efficiency, as measured by the foetal/placental mass ratio, was not altered between WT and mutant conceptuses (Fig. 2c). We saw no indication of lethality in late gestation because the expected Mendelian ratios of WT : TG^PAT^ embryos were observed (Fig. 2c).

To understand the growth retardation phenotype of the TG^PAT^ conceptuses, we looked for evidence of disruption of the major embryonic growth pathway, mediated by IGF1 and IGF2 signalling. Pre-natal growth retardation has been observed following deletion of *Igf1*, *Igf2* and their receptor *Igf1r* (reviewed in Efstratiadis 1998), *Igfbp4* (Ning *et al*. 2006) or overexpression of *Igfbp1* (Ben Lagha *et al*. 2006). Deletion of *Grb10* results in growth enhancement (Charalambous *et al*. 2003). Thus, we measured expression of all these genes in e15.5 embryos and placentae. In the placenta, we observed no changes in the expression of IGF pathway genes (including the placental-specific *Igf2* transcript P0). In addition, expression levels of *Igf1*, I*gf2*, *Igfr1* and *Grb10* did not differ between WT and TG^PAT^ embryos. However, in the TG^PAT^ embryo, *Igfbp1* was elevated and *Igfbp4* expression was reduced, consistent with growth retardation (Fig. 2d).

### TG^PAT^ animals fail to thrive in the early post-natal period, but partially catch up in growth after weaning

The majority of the TG^PAT^ pups died on the day of birth, consistent with previous reports (Schuster-Gossler *et al*. 1996, Steshina *et al*. 2006). Of the remainder only 72% of the transgenic animals survived until weaning at 28 days post-partum (compared with 97% survival of WT littermates; *n* = 15 litters, *P* < 0.05 by Mann–Whitney *U*-test).

**Figure 3:**
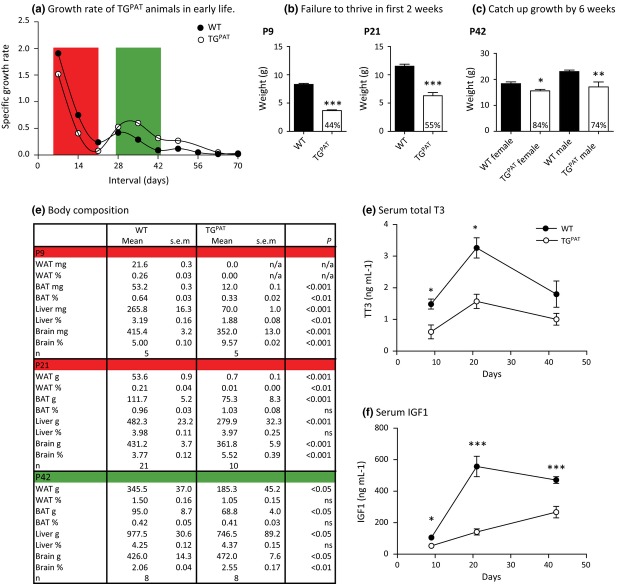
Reduced growth rate of TG^PAT^ animals is associated with reduced serum T3 and IGF1. (a) Specific growth rates calculated from growth measurements of male mice from birth to 70 days of age, *n* = 12 TG^PAT^ and 18 wild-type (WT) littermates, using the following equation: (weight at *T*_2_−weight at *T*_1_)/weight at *T*_1_). The portion of the growth curve highlighted in red indicates a period when mutant growth rate is reduced relative to WT (failure to thrive), and the area highlighted in green indicates an interval when TG^PAT^ exceeds the WT growth rate (catch-up growth). (b) Mean whole body weights at P9 and P21 of WT and TG^PAT^ animals ± SEM (P9, *n* = 5 per genotype; P21 *n* = 21 WT and *n* = 10 TG^PAT^, ***P < 0.001 by Mann–Whitney *U*-test). Number inside the bar indicates percentage WT weight of mutant pups. Sexes are combined. (c) Mean body weight at 6 weeks of WT and TG^PAT^ females and males ± SEM (females *n* = 3–4 per genotype, males *n* = 8 per genotype, *P < 0.05, **P < 0.01 by Mann–Whitney *U*-test). Number inside the bar indicates percentage WT weight of mutant animals. (d) Organ weights and organ weights as percentage body weight in WT and mutant animals at P9, P21 and 6 weeks of age. Sexes are combined at P9 and P21, and at 6 weeks only males are shown. *P*-values are returned from a Mann–Whitney *U*-test of comparison of WT and TG^PAT^ values. (e) Mean serum total T3 levels ± SEM of mice shown in (d). *P < 0.05 by Mann–Whitney *U*-test. (f) Mean serum IGF1 levels ± SEM of mice shown in (d). *P < 0.05, ***P < 0.001 by Mann–Whitney *U*-test.

On the day of birth, TG^PAT^ animals were growth-restricted by 28% (WT 1.58 ± 0.04 g, *n* = 7, TG^PAT^ 1.14 ± 0.03 g, *n* = 13, *P* < 0.001 Mann–Whitney *U*-test). We measured the weights of TG^PAT^ animals and their WT littermates over a 12-week period and then calculated the rate of growth during this interval (Fig. 3a). In the first 3 weeks, the TG^PAT^ animals had a reduced growth rate compared with their WT littermates such that they were growth-retarded by 45% at post-natal day 21 (P21, Fig. 3a,b). After P21, both WT and TG^PAT^ animals displayed a post-pubertal growth spurt that was maximal between P28 and P42. In TG^PAT^ animals, this growth spurt had an increased rate relative to WTs. As a result of this at P42, TG^PAT^ animals had caught up to between 74 and 84% WT weight (Fig. 3a,c).

To more fully understand the growth-restricted phenotype, we examined body composition at two time points, within the failure to thrive phase and after catch-up growth had occurred (Fig. 3d). At P9, all organs tested were significantly smaller in TG^PAT^ animals, both in absolute weight and as a function of body weight, with the exception of the brain which was spared. Abdominal white adipose tissue (WAT) stores failed to develop in TG^PAT^ animals. At P21, brown adipose tissue and liver weight normalized to body weight, and the brain sparing was less marked, but appreciable WAT stores had still failed to develop in the mutant animals and circulating leptin levels were low (WT 3.7 ± 0.4 ng mL^−1^, *n* = 13; TG^PAT^ 1.0 ± 0.1 ng mL^−1^, *n* = 11; *P* < 0.001 by Students' *t*-test). By 6 weeks of age, TG^PAT^ animals had almost completely gained proportionality, with only mild brain sparing, and WAT stores were accumulating to an appropriate mass for body weight.

Previous work has shown that *Dio3* expression in early life has a critical role in programming the future thyroid hormone axis. *Dio3*−/− mice experience peri-natal thyrotoxicosis followed by hypothyroidism due to both impaired central feedback mechanisms and thyroid insufficiency (Hernandez *et al*. 2006, 2007). To test whether there might be an involvement of the thyroid axis in the phenotype of TG^PAT^ mice, we measured T3 levels at various post-natal time points. We saw no evidence of pre-natal thyrotoxicosis in TG^PAT^ mice; instead at P2, mutants were already hypothyroid (TG^PAT^ 0.31 ng mL^−1^, *n* = 7; WT 0.44 ng mL^−1^, *n* = 8; *P* < 0.05 by Mann–Whitney *U*-test). We found that serum T3 was still reduced in mutant animals at P9 and P21, but that hormone levels had begun to normalize by 6 weeks (Fig. 3e). We measured T4 at P9 and 6 weeks and found that thyroxine was also reduced at these stages (P9 WT 1.56 ± 0.83 ng mL^−1^, *n* = 3, TG^PAT^ 0.81 ± 0.67 *n* = 8, ns, P42 WT 4.42 ± 0.11 ng mL^−1^, *n* = 6; TG^PAT^ 2.48 ± 0.18, *n* = 4, *P* < 0.01 Mann–Whitney *U*-test) suggesting a defect in central feedback or thyroid insufficiency similar to *Dio3*^−/−^ mice. We concluded that the growth phenotype broadly followed the level of thyroid deficiency in the mutant animals.

We next asked whether the GH–IGF1 pathway was altered in the TG^PAT^ mutant animals. In contrast to humans, rodent GH is activated post-natally at approx. P10, and GH pathway mutants are not growth-restricted until after this stage (Lupu *et al*. 2001). In contrast, IGF1 mutation affects the growth rate continuously during development at all growth stages. However, GH secretion causes a surge in IGF1 levels around P21, causing the pubertal growth spurt (Mathews *et al*. 1988). Thyroid hormone signalling is known to promote pituitary GH production (Gothe *et al*. 1999), yet it may also directly influence IGF1 levels because TH pathway mutants are growth-restricted prior to P10 (Fraichard *et al*. 1997, Gothe *et al*. 1999, Hernandez *et al*. 2006). We found a reduction in circulating IGF1 levels in TG^PAT^ animals at all three developmental stages examined, including prior to induction of GH secretion at P9 (Fig. 3f). In support of a GH-independent mechanism for altered IGF1 levels in the mutants, we could not detect GH in serum of WT or mutant animals at P9 (data not shown). However, we could detect GH at 6 weeks, and there was a trend for secretion to be reduced in the TG^PAT^ animals, although levels were extremely variable (WT 6.0 ± 2.4 ng mL^−1^; TG^PAT^ 2.7 ± 1.7 ng mL^−1^, *P* = 0.27, Mann–Whitney *U*-test).

We concluded that the growth retardation in TG^PAT^ animals was a likely consequence of both the known mechanism of altered pituitary GH-mediated IGF1 secretion and an unknown peri-natal mechanism of IGF1 regulation that is independent of GH.

### TG^PAT^ adults are small with a mild impairment in glucose homeostasis

**Figure 4:**
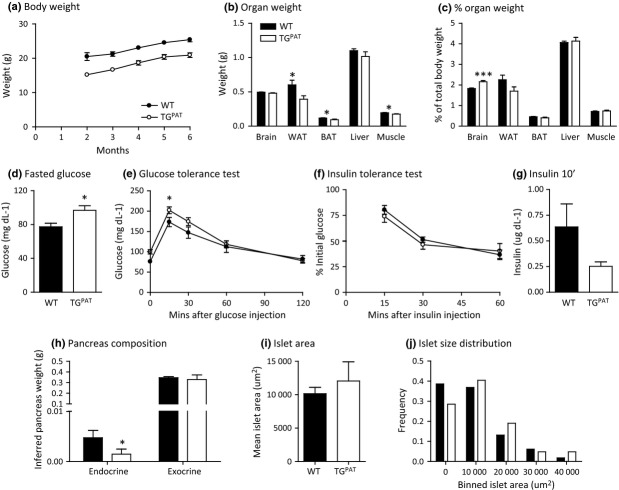
Reduction in body size and impairments in glucose homeostasis in TG^PAT^ adults. Growth and glucose homeostasis were studied in a cohort of 19 wild-type (WT) and 18 TG^PAT^ females at 6 months of age. (a) Animals were weighed weekly over 6 months, and mean weight each month between WT and TG^MAT^ animals was compared by anova (*P* < 0.001 overall and *P* < 0.01 at each time point by Bonferroni's *post hoc* test). (b) Organ weight at 6 months of age and presented as a percentage of total weight, (c). (d) Serum glucose after an overnight fast (approx. 16 h). TG^PAT^ have elevated fasting glucose compared by Students' *t*-test (*P < 0.05). (e) Glucose tolerance test. Six month-old animals were injected with glucose, and glucose clearance was measured over a 2-h period. TG^PAT^ animals cleared glucose from the blood significantly more slowly that their WT littermates (time points compared by Students' *t*-test, *P < 0.05). (f) Insulin tolerance test. Insulin was injected into 6 month-old animals, and glucose clearance was measured over a 1-h period. (g) Serum insulin 10 min after a glucose challenge. (h) Endocrine (islet) and exocrine pancreatic weights in WT (*n* = 8) and TG^PAT^ (*n* = 6) females at 6 months of age. TG^PAT^ animals have reduced endocrine mass compared with WT littermates (Mann–Whitney *U*-test, *P* < 0.05). (i) Mean area in WT and mutant pancreas, as mentioned above. Average islet area per animal were calculated by measuring multiple islets, and the graphed value represents the mean of the animal averages by genotype. (j) Frequency distribution of islet areas for all animals by genotype (WT *n* = 118 islets; TG^PAT^ *n* = 42 islets).

From 2 months of age, TG^PAT^ body weight stabilized to approx. 80% WT mass (2 months, 75%; 3 months, 78%; 4 months, 81%; 5 months, 83%; 6 months, 83% relative to WT, *P* < 0.001 by one-way anova, Fig. 4a). Adult organ weights were also reduced in size largely in proportion to total body weight, except the brain which was relatively larger in the mutants (Fig. 4b,c).

At 6 months of age, TG^PAT^ mice exhibited mild fasting hyperglycaemia (Fig. 4d), which led us to ask whether glucose homeostasis was impaired. When we performed intraperitoneal GTTs at 6 months, we found a slight reduction in glucose clearance by mutant animals (Fig. 4e). Peripheral insulin sensitivity was not different between WT and TG^PAT^ mice (Fig. 4f). To test whether insulin secretion was affected in the mutants, we measured serum insulin in response to a glucose challenge. We found a tendency for reduced serum insulin in the TG^PAT^ mice, but this was not statistically significant (*P* = 0.12).

Alterations to IGF signalling are known to cause defects in endocrine pancreas development such that islet mass is compromised and insulin secretion is reduced (Withers *et al*. 1999). Moreover, perturbations to the TH signalling pathway (including ablation of *Dio3*) causes alterations in endocrine mass and acute-phase insulin secretion (Taguchi *et al*. 2010, Medina *et al*. 2011). We explored the glucose homeostasis phenotype of TG^PAT^ animals further by asking whether pancreatic morphology was affected by the mutation. Endocrine pancreas mass was significantly reduced in TG^PAT^ animals, but exocrine mass was unaffected (Fig. 4h). The reduction in endocrine mass was not a result of decreased islet size, because the mean islet area was not reduced, and islet size distribution showed shift towards increased islet size in the mutants (Fig. 4i,g). We concluded that the reduced endocrine mass observed in the TG^PAT^ pancreas was probably due to a reduction in islet number. Furthermore, the mild defect in glucose clearance that we observed in TG^PAT^ animals is consistent with defect in insulin secretion as a result of a compromised endocrine pancreas.

### Alterations to the GH/TH axis are transient, because TG^PAT^ adults have normal serum TH and IGF1

We measured serum hormone levels in WT and TG^PAT^ mice at 6 months of age (Table 1). Serum levels of total T3, total T4, IGF1 and leptin did not differ between the genotypes. We concluded the adult impairment to glucose homeostasis observed in TG^PAT^ mice was likely to be caused by a developmental defect in endocrine pancreatic development.

**Table 1:** Serum parameters in 6-month-old female mice

	WT			TG^PAT^			*P*
	Mean	SE	*n*	Mean	SE	*n*	
Adult females							
tT3 (ng mL^−1^)	2.66	0.30	6	3.10	0.42	6	ns
tT4 (ng mL^−1^)	5.17	0.12		6.05	0.23		ns
IGF1 (ng mL^−1^)	644	63		521	77		ns
Leptin (ng mL^−1^)	4.58	0.88		3.19	0.51		ns

WT, wild-type.

*P*-values were a result of a Mann–Whitney *U*-test comparing genotypes; all tests were not significant (ns).

## Discussion

Insertion of a LacZ transgene upstream of a control element at the mouse *Gtl2* promoter prevents the methylation of this region *in cis*. This epigenetic lesion is associated with misexpression of neighbouring genes such that non-coding RNA expression is elevated, and levels of *Dlk1*, *Rtl1* and *Dio3* transcripts are reduced.

We observed three key phenotypes in TG^PAT^ mice as a consequence of this transgene insertion: embryonic growth retardation, reduced post-natal growth rate and impaired adult glucose homeostasis. We show for the first time that the chromosome 12 cluster in the mouse is an imprinted locus that modulates the IGF signalling pathway and propose that the phenotypes of TG^PAT^ mice may be attributable to impaired IGF signalling.

TG^PAT^ mice are 10–15% smaller than their WT littermates in late gestation, and this growth deficit is observed in the embryo and in the placenta. We saw no gross phenotypic differences between the genotypes, which led us to conclude that the weight reduction was a growth phenotype rather than an alternative developmental defect. Altered expression of most IGF pathway genes can cause changes in embryonic weight (Efstratiadis 1998). We measured gene expression of IGF pathway components in late gestation placenta and embryo and found that in the embryo, *Igfbp1* expression was elevated and *Igfbp4* expression was reduced. These changes are consistent with growth effects reported in mouse deletion or overexpression models (Ben Lagha *et al*. 2006, Ning *et al*. 2006). Interestingly, *Igfbp1* overexpression has also been linked to growth retardation both in mouse foetal programming models (Woodall *et al*. 1996) and in human FGR (Wang *et al*. 1991). In addition, both *Igfbp1* and *Igfbp4* have been demonstrated to be regulated by thyroid hormones (Demori *et al*. 1997). We observed a significant reduction in D3 activity in TG^PAT^ embryos, and therefore, foetal tissues would be expected to experience an elevated local TH level. However, we cannot rule out regulation by other chromosome 12-imprinted genes, because *Dlk1* deletion and *Rtl1* deletion models also exhibit mild growth retardation (Moon *et al*. 2002, Sekita *et al*. 2008). However, growth retardation and late embryonic lethality in *Rtl1*-deleted mice are attributed to a placental defect (Sekita *et al*. 2008), and we saw no change in *Rtl1* expression in TG^PAT^ placentae.

Paradoxically, despite reduced D3 activity during embryogenesis that would be expected to reduce T3 clearance and cause hyperthyroidism, we found that TG^PAT^ animals had reduced circulating T3 from shortly after birth. *Dio3*^−/−^ mice are also hypothyroid, but this follows an early period of elevated T3 (Hernandez *et al*. 2006). The differences between these two models may be explained by the difference in perturbation of D3 (TG^PAT^ has approx. 50% WT levels, whereas *Dio3*^−/−^ has complete ablation) or additional involvement of other chromosome 12-imprinted genes in the TH phenotype. However, both models have in common pre-and post-natal growth retardation.

TG^PAT^ mice were born small and failed to gain weight at the normal rate in the immediate post-natal period. This early failure to thrive period was accompanied by brain sparing and a failure to accumulate appropriate WAT stores. The low growth rate was associated with reduced serum IGF1 and T3 in mutant animals.

TH is known to regulate pituitary GH secretion, and mouse models of TH deficiency are commonly assumed to have growth retardation primarily due to impaired GH signalling (Kindblom *et al*. 2001). However, several models of TH deficiency, including the *Dio3*^−/−^ model, exhibit growth retardation in the embryonic and early post-natal period (Gothe *et al*. 1999, Hernandez *et al*. 2006) when growth and IGF1 secretion are known to be GH independent (Lupu *et al*. 2001). Our work provides an additional example of hypothyroidism, low IGF1 and growth retardation, which is not GH dependent, and highlights need for further research into TH pathway regulation of IGF1.

After weaning, TG^PAT^ mice exhibit catch-up growth and largely regain proportionality. We could not discover the cause for this catch-up, but it was associated with normalization of T3 and IGF1 levels such that in the adult mutants, levels of these hormones were normal. Adult mutant mice were mildly growth-retarded with proportional organ mass. However, we observed mild fasting hyperglycaemia and impaired glucose tolerance in TG^PAT^ animals at 6 months of age. As we saw a small perturbation in glucose-stimulated insulin secretion, we investigated the cell composition of the mature pancreas in mutant mice. TG^PAT^ animals had a reduction in endocrine pancreas mass, which was attributed to reduced islet number.

Global deletion of the *Igf1* receptor in mice compromises pancreatic development such that in late gestation, endocrine cells fail to form spherical islet structures and endocrine cell number is reduced. Milder disruptions to the IGF signalling pathway (caused by combinations of *Igf1r*, *Irs1* or *Irs2* deletion or haploinsufficiency) result in disruption to the proliferation/apoptotic balance in beta cells and net loss of insulin-producing cells (Withers *et al*. 1999). Beta cell-specific ablation of the *Igf1* receptor does not retard endocrine pancreas development, but impairs acute-phase insulin release because of a defect in glucose sensing (Kulkarni *et al*. 2002). Combined, these data suggest that the IGF pathway has a dual role in beta cell function, both during the development of the endocrine pancreas and continuously in the beta cell to regulate genes responsible for glucose sensing.

Models of disruption to the TH pathway have strikingly similar pancreatic phenotypes to IGF pathway mutants, suggesting that they are secondary to IGF pathway disruption. Hypothyroid growth-retarded mice (Taguchi *et al*. 2010) have a defect in responsiveness to thyroid-stimulating hormone, which causes reduced circulating thyroid hormone levels. Mutant mice have impaired glucose tolerance, elevated fasting glucose but normal peripheral insulin sensitivity. The hyperglycaemia was found to be a defect in acute-phase insulin secretion by the beta cell. Interestingly, the phenotype could not be rescued by restoring circulating T3, suggesting there may be a developmental defect. IGF1 levels have not been reported in these animals. This phenotype is very similar to *Dio3*^−/−^ mice, which are hypothyroid due to peri-natal thyrotoxicosis (Hernandez *et al*. 2006). Small *Dio3*^−/−^ adult mice have defects in glucose homeostasis due to a failure of acute-phase insulin secretion along with a reduction in beta cell mass. Again, the defects are not a result of systemic hypothyroidism because adult induction of hypothyroid state by methimazole administration did not cause defects in glucose clearance. The authors propose that local D3 acting in the beta cell and/or a developmental cause may explain the phenotype (Medina *et al*. 2011). We favour the latter explanation because the activity of D3 in the beta cell is extremely low, at least two orders of magnitude lower than other known sites of D3 action (brain and placenta).

We propose that TG^PAT^ mice experience hypothyroidism and IGF1 deficiency during a critical developmental window, such that they are growth-retarded, fail to accumulate fat in the early post-natal period and have a long-term impairment to glucose homeostasis due to a failure of endocrine pancreas development. The gene expression changes in TG^PAT^ mice are similar to those observed in cases of Temple syndrome. Further study of IGF pathway involvement in the FGR associated with Temple syndrome may aid early diagnosis and treatment of affected patients.

## Conflict of interest

The authors declare no conflicts of interest.

## 

The authors would like to thank Chris Angiolini and Scott Curran for technical assistance, Professor Graham Burton for expert advice on stereological methods and Professor Achim Gossler and Dr Karin Schuster-Gossler for the mice. Work was funded by EU FP7 Grant – EpigeneSys Network of Excellence and by the MRC.

## Supporting Information

Table S1 Primer sequences for real time quantitative PCR.
