# Supplementary material for: Perturbations to the IGF1 growth pathway and adult energy homeostasis following disruption of mouse chromosome 12 imprinting
Source: Acta Physiol (Oxf). 2013 Sep 18;210(1):174–87. doi: 10.1111/apha.12160 (PMC3992899; doi:10.1111/apha.12160)
Supplement: Table S1 — Primer sequences for real time quantitative PCR. [file apha0210-0174-sd1.docx]

| **primer** | **Forward** | **Reverse** | **Ref (if applicable)** |
| --- | --- | --- | --- |
| Dlk1 | GAAAGGACTGCCAGCACAAG | CACAGAAGTTGCCTGAGAAGC | Charalambous et al., 2012) |
| Gtl2 | GGACACACGGACACAGACA | TGTCCCACAGGAAATGTGCAA | Charalambous et al., 2012) |
| Meg9/Mirg | GGACATCCATGGGAGAGCTA | AGAGCAGAAACCCCTCCTTC | Charalambous et al., 2012) |
| Igf1 | GCTCCATTAGACACACCCTTTC | ATGCCACAGATGGAGTCAGGT |  |
| Igf2 | CGCTTCAGTTTGTCTGTTCG | GGGGTGGCACAGTATGTCTC | Radford et al. 2012 |
| Igf2P0 | GCATCCCCGGTCCTCTTTAT | GCTCTGGCTGGACGAGAAGT | Charalambous et al., 2010 |
| Igf1r | CCGAACCCTTAACTGACATGG | AGGGAAGGCAGAGGAGAGAAA |  |
| HPRT | CAGGCCAGACTTTGTTGGAT | TTGCGCTCATCTTAGGCTTT | Charalambous et al., 2012 |
| Igfbp1 | CTGCCAAACTGCAACAAGAA | GACCCAGGGATTTTCTTTCC |  |
| Igfbp4 | GAGATCGGAGCAAGATGAAGA | ATGGGGATGATGAAGAGGTC |  |
| Grb10 | TGCACCACTTCTTGAGGATG | ACCAGTGAGCTCCGGAAATG | Radford et al.2012 |

Supplementary Table 1. Primer sequences for real time quantitative PCR.
